# Clinical and CT body-composition model for major pathological response to neoadjuvant tislelizumab, albumin-bound paclitaxel, and nedaplatin in esophageal squamous cell carcinoma

**DOI:** 10.3389/fonc.2026.1859690

**Published:** 2026-10-30

**Authors:** Huiran Xu, Wei Zheng, Shumei Chen, Suying Wu

**Affiliations:** 1 Department of Radiology, The First Hospital of Putian City, Putian, Fujian, China; 2 Department of Pathology, The First Hospital of Putian City, Putian, Fujian, China

**Keywords:** esophageal squamous cell carcinoma, major pathological response, neoadjuvant immunochemotherapy, osteoporosis, sarcopenia

## Abstract

**Background:**

Sarcopenia and osteoporosis are linked to poor treatment outcomes and are significantly underdiagnosed in esophageal cancer patients. Despite the promising efficacy of neoadjuvant immunochemotherapy (NACI) in esophageal squamous cell carcinoma (ESCC), predicting treatment response remains a major clinical hurdle.

**Objective:**

This study aimed to evaluate the associations of sarcopenia and osteoporosis on pathological response and to develop a both clinical and computed tomography (CT)-based predictive model for NACI outcomes in ESCC patients.

**Methods:**

A retrospective analysis was conducted in 179 patients with ESCC who underwent radical resection after neoadjuvant tislelizumab, albumin-bound paclitaxel, and nedaplatin. Within each of 500 stratified bootstrap resamples, five-fold cross-validation was used to determine the optimal LASSO penalty parameter, followed by Firth logistic regression. Aggregated out-of-bag predictions underwent five-fold cross-fitted recalibration. Model discrimination, calibration, and clinical utility were evaluated using the AUC, accuracy, sensitivity, specificity, Brier score, calibration intercept and slope, Hosmer–Lemeshow test, and decision curve analysis.

**Results:**

Major pathological response (MPR) rates were 70.0%, 40.0%, 22.0%, and 8.3% in the control, osteoporosis-only, sarcopenia-only, and osteosarcopenia groups, respectively. In multivariable Firth logistic regression, lower tumor grading, absence of comorbidities, absence of lymphovascular invasion, absence of sarcopenia, and a higher intermuscular adipose tissue (IMAT) area percentage were associated with a greater likelihood of achieving MPR, whereas osteoporosis was not associated with MPR (P = 0.130). The cross-fitted recalibrated model achieved an AUC of 0.885 (95% CI, 0.835–0.934), with 0.818 accuracy, 0.840 sensitivity, and 0.800 specificity at a threshold of 0.405. Calibration was satisfactory (Brier score, 0.136; intercept, −0.009; slope, 0.960; Hosmer–Lemeshow P = 0.219), and decision curve analysis showed clinical net benefit across thresholds of 0.08–0.80.

**Conclusion:**

Absence of sarcopenia and a higher IMAT area percentage were associated with a greater likelihood of achieving major pathological response. The combined clinical and CT-based model showed good internally validated performance.

## Introduction

Esophageal cancer is the seventh most diagnosed cancer and the sixth leading cause of cancer-related death worldwide. China accounts for more than half of global esophageal cancer cases and deaths, with esophageal squamous cell carcinoma (ESCC) representing approximately 90% of cases (1, 2). Neoadjuvant chemoradiotherapy is a standard treatment for locally advanced esophageal cancer, although treatment responses vary, and perioperative risks remain. More recently, immunotherapy has reshaped the neoadjuvant treatment landscape for ESCC. Neoadjuvant camrelizumab combined with paclitaxel and nedaplatin achieved major pathological response (MPR) and pathological complete response (pCR) rates of 45.2% and 27.4%, respectively (3). Two other studies using three cycles of neoadjuvant immunochemotherapy (NACI) reported MPR rates of 69.2% and 72.0% with pembrolizumab plus doublet chemotherapy and tislelizumab plus carboplatin and albumin-bound paclitaxel, respectively (4, 5).

Pathological response is an important measure of short-term efficacy after neoadjuvant therapy, and the achievement of MPR or pCR is associated with improved long-term outcomes. MPR is generally defined as no more than 10% residual viable tumor in the primary lesion and regional lymph nodes, whereas pCR indicates the absence of viable tumor cells (6). However, the factors associated with variation in pathological response to NACI among patients with ESCC remain incompletely understood.

Accumulating evidence indicates that sarcopenia and osteoporosis are associated with adverse clinical outcomes in several malignancies, including esophageal cancer (7–9). Sarcopenia is characterized by reduced skeletal muscle mass and function, whereas osteoporosis is defined by decreased bone mineral density (BMD). Both conditions are associated with falls, fractures, functional impairment, mortality, and reduced quality of life. Low BMD may also be associated with the progression of sarcopenia (10). In patients with cancer, subcutaneous and visceral adipose tissue volumes have been associated with sarcopenia and recurrence-free survival (11). Osteosarcopenia, defined as the coexistence of sarcopenia and osteoporosis, has also emerged as a potential prognostic factor in several cancers (12, 13). However, the relationships of sarcopenia, osteoporosis, osteosarcopenia, and other CT-derived body-composition parameters with MPR after NACI in ESCC remain poorly understood.

Therefore, this study aimed to evaluate the associations of sarcopenia, osteoporosis and CT-derived body-composition parameters with the achievement of MPR in patients with ESCC treated with neoadjuvant tislelizumab, albumin-bound paclitaxel, and nedaplatin. We also developed and internally validated an integrated clinicopathological and CT body-composition model for classifying MPR status.

## Material and methods

### Patients and eligibility criteria

This retrospective study screened patients with ESCC treated at our hospital between March 2019 and April 2025. Patients were eligible if they (1): had histologically confirmed ESCC (2); received the designated NACI regimen comprising tislelizumab, albumin-bound paclitaxel, and nedaplatin (3); subsequently underwent radical surgical resection; and (4) had pretreatment CT images and postoperative pathological assessment available. Patients were excluded if they (1): received other neoadjuvant antitumor regimens (2); had concurrent malignancies (3); had incomplete blood biomarker data; or (4) had inadequate CT image quality for quantitative assessment. After applying these criteria, 179 patients were clinically eligible (**Figure 1**).

**Figure 1:**
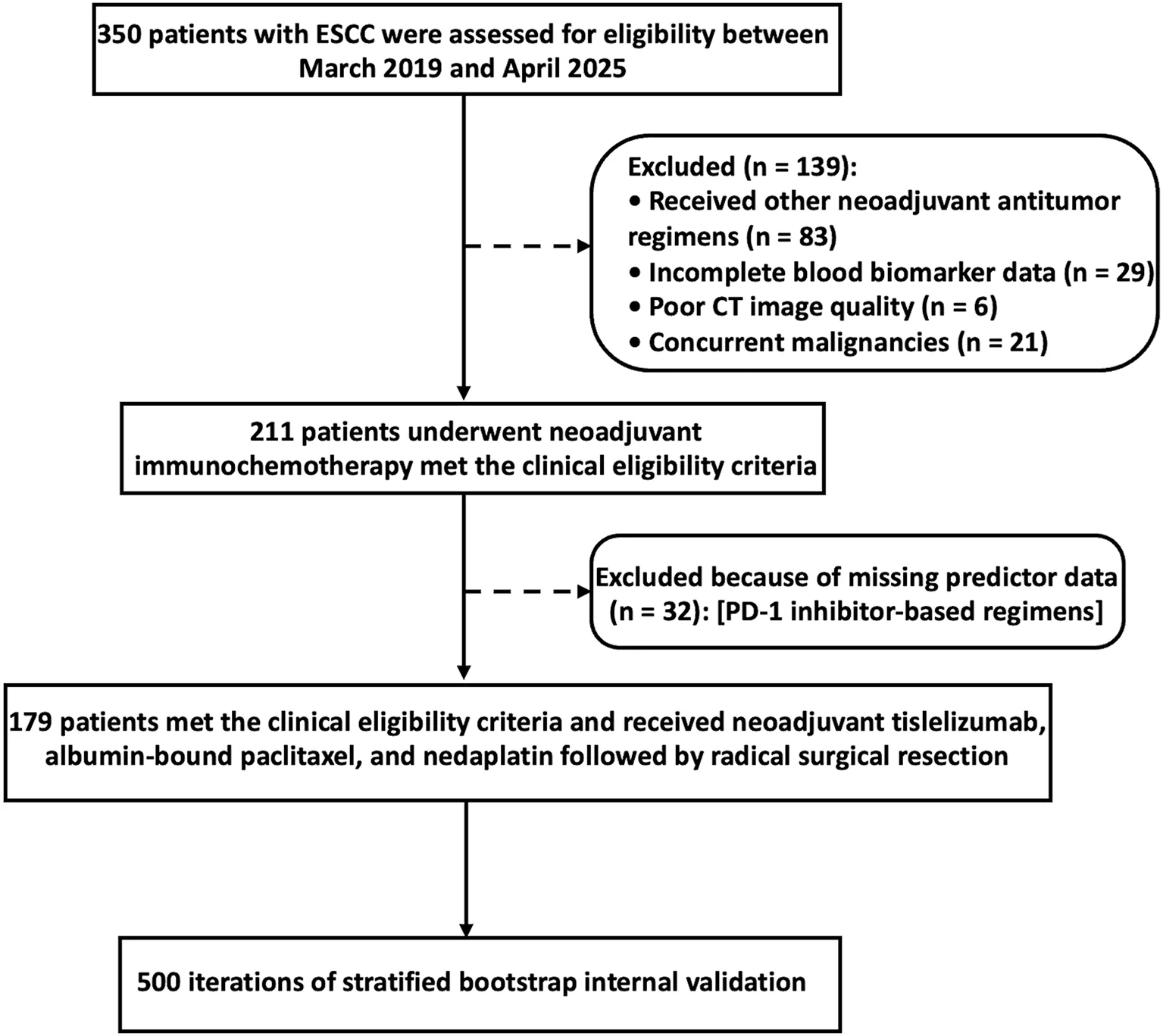
Patient selection.

Repeated stratified bootstrap resampling was performed 500 times using the entire cohort. In each iteration, model development was conducted within the bootstrap training sample, and predictions were obtained exclusively for out-of-bag patients. Patient-level out-of-bag probabilities were averaged across iterations and subsequently recalibrated using five-fold cross-fitting. Discrimination, overall prediction error, calibration, and clinical utility were evaluated using the pooled cross-fitted predictions from all patients. Patients were staged using the esophageal cancer TNM staging system (14).

In this study, the NACI regimen consisted of tislelizumab (200 mg), albumin-bound paclitaxel (300 mg), and nedaplatin (100 mg). All patients received the designated NACI regimen intravenously on day 1 of each 21-day cycle. Treatment consisted of 2 to 4 cycles, tailored to individual patient response. Radical surgical resection was performed upon completion of the neoadjuvant therapy.

### Pathologic evaluation

To determine the MPR status, two pathologists, with 10 and 15 years of experience, evaluated the proportion of residual tumor cells present in both the primary tumor and lymph nodes in H&E-stained slides using high-power microscopy. In this study, MPR was defined as the proportion of residual tumor cells in the primary tumor and lymph nodes being ≤10%, with residual tumor cells >10% classified as non-MPR. Any discrepancies were resolved through discussion and consensus.

### Clinicopathological information

For all enrolled patients, comprehensive clinicopathological information was retrospectively extracted from the electronic medical record system. These data encompassed baseline characteristics, core indicators of blood routine and blood biochemistry. Additionally, we documented and analyzed the pathological characteristics obtained from radical surgically resected specimens.

Prior to NACI, peripheral blood samples were drawn. Researcher computed the prognostic nutrition index (PNI) as albumin (g/dL) × 10 + 0.005 × lymphocyte count; neutrophil-lymphocyte ratio (NLR) as neutrophil count/lymphocyte count; platelet-lymphocyte ratio (PLR) as platelet count/lymphocyte count; and lymphocyte-monocyte ratio (LMR) as lymphocyte count/monocyte count, as indicators of systemic inflammation and nutritional status.

### CT scanning

CT scans were performed using a SOMATOM Force multi-slice spiral CT scanner (Siemens, Germany). With patients in a supine position, cranio-caudal scanning was conducted after determining the scanning range through scout images. Images were reviewed using both soft tissue and bone windows. CT scans were performed with a tube voltage of 120 kV using CARE Dose4D modulation, a rotation time of 0.5 s, and a pitch of 0.8. The reconstructed images had a 350 mm × 350 mm field of view, a 512 × 512 matrix, and consecutive 1.0 mm slice thickness.

### CT image evaluation

The study utilized CT images in DICOM format sourced from the imaging archive system. Body-composition and bone-density parameters were quantified from preoperative non-contrast CT images acquired within one month before radical surgical resection: BMD, volume/area percentages of subcutaneous adipose tissue (SAT), volume/area percentages of intermuscular adipose tissue (IMAT), volume/area percentages of visceral adipose tissue (VAT), psoas muscle index (PMI) and total psoas area (TPA). CT images were assessed with a specialized image analysis software (Bones, Shenzhen, China). TPA was assessed at the third lumbar vertebra (L3) on axial CT slices, with PMI calculated as PMI = TPA (cm²)/height (m²). Sarcopenia was defined by gender-specific PMI thresholds:< 6.36 cm²/m² for men and< 3.92 cm²/m² for women, in accordance with established criteria (15, 16). Measurement of the fat-related percentage were measured on CT sectional imaging at the second and third intervertebral disc levels. Two experienced attending radiologists manually delineated the regions of interest (ROI) on the corresponding CT slices. Any discrepancies were reviewed and adjudicated by a third senior radiologist. The software then automatically generated three-dimensional volumes of interest (VOI). Using the analysis system tool QCT, the fat-related features of each patient’s VOI were extracted. BMD was derived from the mean pixel density measured within an elliptical ROI at the T11 vertebral level. Osteoporosis was defined as an absolute BMD of less than 80 mg/cm^3^. Osteosarcopenia was considered present when sarcopenia and osteoporosis coexisted. The imaging findings are shown in **Figure 2**.

**Figure 2:**
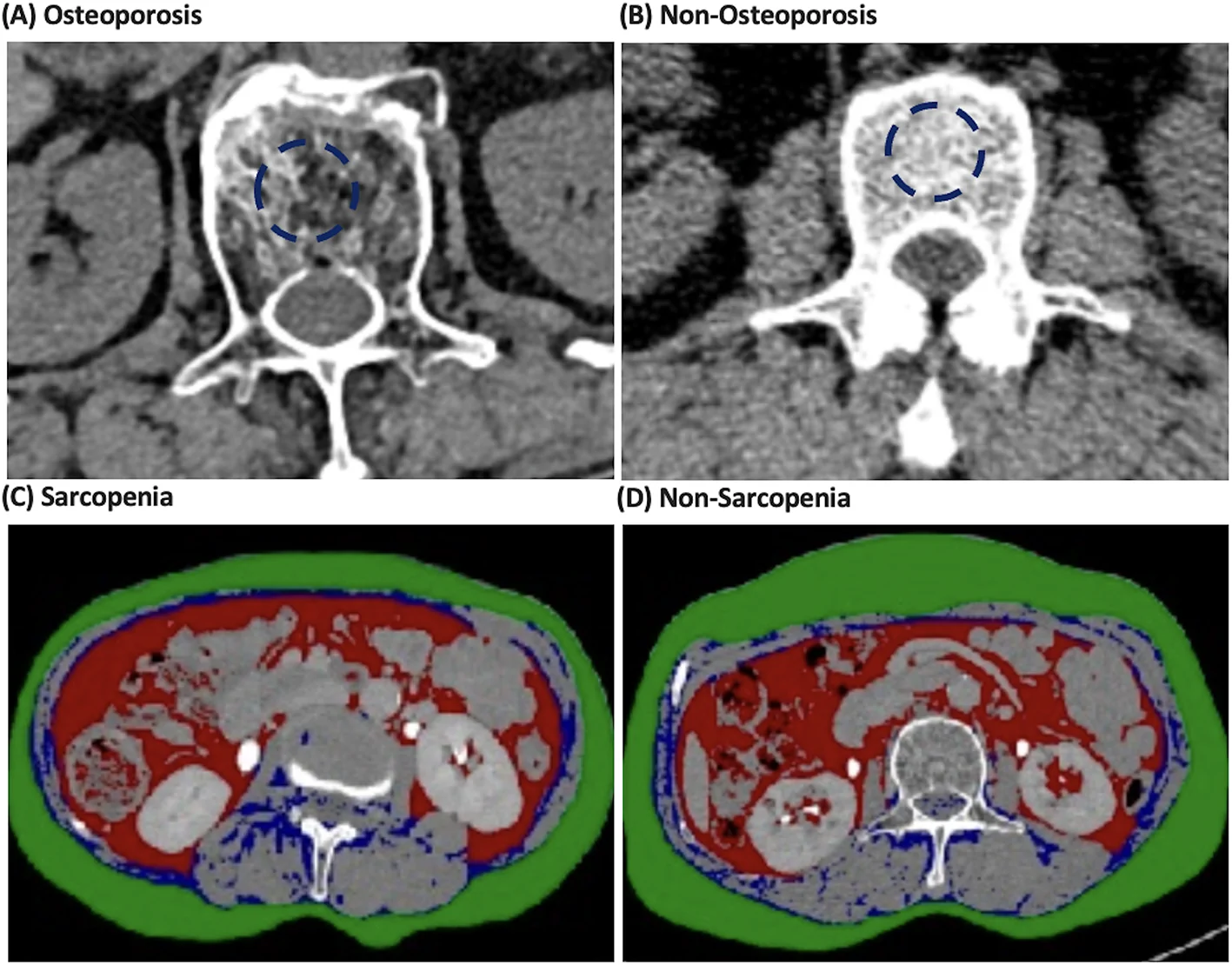
Representative CT images for osteoporosis and sarcopenia. **(A, B)** Representative axial CT images from patients with and without osteoporosis, respectively. The blue dashed circles indicate the vertebral trabecular regions of interest used for bone mineral density measurement. **(C, D)** Representative body-composition segmentation images from patients with and without sarcopenia, respectively. Green represents subcutaneous adipose tissue, red represents visceral adipose tissue, and blue represents intermuscular adipose tissue.

### Statistical analysis

The statistical computing environment R, version 4.1.3, was utilized for analyses. Data handling, regression modeling (utilizing logistf and glmnet), validation (with rms and rmda), and visualization (using ggplot2 and gridExtra) were performed with the respective packages. Normally distributed continuous variables were expressed as mean ± standard deviation and compared using Student’s t-test. Non-normally distributed variables were expressed as median (interquartile range) and compared using the Wilcoxon rank-sum test. Categorical variables were expressed as counts and percentages and compared using Pearson’s χ² test. Between-group comparisons were descriptive. Predictor selection followed the LASSO–Firth modelling procedure, rather than inclusion based solely on significant between-group differences.

A nested internal-validation procedure was implemented. In each of 500 stratified bootstrap iterations, five-fold cross-validation was performed within the bootstrap training sample to determine the optimal LASSO penalty parameter (*λ*). Predictors with non-zero coefficients were subsequently entered into a Firth logistic regression model, which was then applied to the corresponding out-of-bag patients to estimate their probabilities of achieving MPR. Multiple out-of-bag predictions for each patient were averaged and used to assess model discrimination, classification performance, and calibration. After internal validation, the final LASSO–Firth model was refitted using the entire cohort and a nomogram based on the final model was constructed. P< 0.05 indicating statistical significance.

## Result

### Baseline clinicopathological profiles

**Table 1** compares the demographic and preoperative clinicopathological characteristics in ESCC individuals based on their MPR status. The average BMI and weight were 21.27 ± 2.49 kg/m^2^ and 57.35 ± 7.96 kg, respectively. Among all, MPR was achieved in 82 (46%) patients, while 97 (54%) patients failed to remit. In the MPR group, tumor size (P = 0.04), SCC antigen (P = 0.04), NLR (P = 0.04), and the proportion of patients with lymphovascular invasion (P = 0.00) were lower, whereas weight (P = 0.00), the proportion of patients with tumor grade < G2 (P = 0.00), and the proportion with a prognostic nutritional index ≥ 50 (P = 0.03) were higher than in the major pathological non-response group.

**Table 1:** Clinicopathological characteristics according to MPR status.

Variable	MPR achieved (n = 82)	MPR not achieved (n = 97)	P value
Age, years	67 (62–72)	67 (62–72)	0.84^b^
Sex, male/female, n	64/18	66/31	0.13^c^
Height, cm	164.0 ± 7.6	164.3 ± 6.8	0.78^a^
Weight, kg	59.05 ± 7.59	55.90 ± 8.02	0.00^a^*
BMI, kg/m^2^	21.2 ± 2.41	21.3 ± 2.59	0.79^a^
Comorbidities, present/absent, n	35/47	56/41	0.04^c^*
ASA-PS,<3/≥ 3, n	48/34	60/37	0.65^c^
Tumor size, mm^2^	2.81 (1.28, 5.06)	4.61 (2.37, 13.05)	0.04^b^*
Tumor stage, T<3/T≥ 3, n	48/34	60/37	0.65^c^
Tumor grading, G<2/G≥ 2, n	70/12	54/43	0.00^c^*
Lymph node metastasis, present/absent, n	48/34	68/29	0.11^c^
LVI, present/absent, n	3/79	32/65	0.00^c^*
CEA, ng/ml	2.68 (1.65, 5.10)	2.76 (1.97, 4.24)	0.52^b^
SCC antigen, ng/mL	1.25 (0.87, 2.37)	2.02 (0.92, 7.44)	0.04^b^*
CA125, U/ml	11.59 (8.63, 22.74)	13.10 (8.46, 33.07)	0.67^b^
PNI, ≥50/<50, n	11/71	4/93	0.03^c^*
NLR, ≤2/>2, n	24/58	16/81	0.04^c^*
PLR, ≥ 150/<150, n	53/29	66/31	0.63^c^
LMR, ≤4/>4, n	54/28	63/34	0.90^c^
Albumin, g/L	34.60 (31.48, 38.93)	34.80 (31.65, 38.65)	0.69^b^
Triglycerides, mmol/L	1.08 (0.82, 1.58)	1.16 (0.76, 1.78)	0.73^b^
Total Cholesterol, mmol/L	4.34 (3.63, 5.08)	4.28 (3.50, 5.14)	0.83^b^

BMI, body mass index; ASA-PS, American Society of Anesthesiologists Physical Status; LVI, lymphovascular invasion; CEA, carcinoembryonic antigen; SCC, squamous cell carcinoma; CA125, carbohydrate antigen 125; PNI, prognostic nutritional index; NLR, neutrophil-to-lymphocyte ratio; PLR, platelet-to-lymphocyte ratio; LMR, lymphocyte-to-monocyte ratio

^a^ Student’s t-test.

^b^ Wilcoxon rank-sum test.

^c^ Pearson’s χ² test. *P< 0.05.

### Correlation between major pathological response and musculoskeletal characteristics

**Table 2** presents and compares the primary musculoskeletal imaging characteristics of the patients based on their MPR status. In this study, 39 patients (21.7%) met the criteria for osteoporosis, 74 (41.3%) for sarcopenia, and 24 (13.4%) for osteosarcopenia. MPR was achieved in 63 of 90 patients (70.0%) in the control group, 6 of 15 (40.0%) in the osteoporosis-only group, 11 of 50 (22.0%) in the sarcopenia-only group, and 2 of 24 (8.3%) in the osteosarcopenia group (**Table 3**). MPR achievement rates differed significantly among the four groups (Pearson χ² = 46.416, df = 3, P< 0.001). In unadjusted binary logistic regression with MPR achievement coded as the positive outcome, osteosarcopenia (OR = 0.12, P< 0.05) and osteoporosis alone (OR = 0.29, P< 0.05) were associated with lower odds of achieving MPR than the control condition. The sarcopenia–osteoporosis interaction was not significant (OR = 1.05, P = 0.96), although a potential interaction cannot be excluded because of the limited sample size. After Bonferroni correction, the sarcopenia-only and osteosarcopenia groups remained significantly less likely to achieve MPR than the control group, whereas the osteoporosis-only group did not differ significantly from either the control or sarcopenia-only group (**Figure 3**).

**Table 2:** Comparison of CT-derived body-composition parameters according to MPR status.

Variable	MPR achieved (n = 82)	MPR not achieved (n = 97)	P value
Sarcopenia, present/absent, n	13/69	61/36	0.00*
Osteoporosis, present/absent, n	8/74	32/65	0.00*
BMD, mg/cm^3^	129.40 (98.46, 161.50)	109.30 (71.98, 147.05)	0.03*
SAT volume, %	49.76 ± 10.76	43.12 ± 12.40	0.00*
IMAT volume, %	22 (18, 27)	20 (17, 24)	0.04*
VAT volume, %	44.95 ± 13.36	39.66 ± 15.48	0.02*
SAT area, %	49.33 ± 10.71	42.60 ± 12.27	0.00*
IMAT area, %	22 (17, 26)	20(16, 24)	0.03*
VAT area, %	45.16 ± 13.68	39.42 ± 15.92	0.01*
TPA, cm²	17.13 (10.23, 18.59)	13.42 (8.96, 19.42)	0.34
PMI, cm²/m²	6.38 (4.08, 6.56)	4.87 (3.45, 7.29)	0.43

MPR, major pathological response; BMD, bone mineral density; SAT, subcutaneous adipose tissue; IMAT, intermuscular adipose tissue; VAT, visceral adipose tissue; TPA, total psoas area; PMI, psoas muscle index. *P< 0.05.

**Table 3:** Major pathological response achievement according to sarcopenia and osteoporosis status.

Group	n	MPR achieved, n (%)	MPR not achieved, n (%)
Neither sarcopenia nor osteoporosis	90	63 (70.0%)^a^	27 (30.0%)
Sarcopenia only	50	11 (22.0%)^b^	39 (78.0%)
Osteoporosis only	15	6 (40.0%)^ab^	9 (60.0%)
Osteosarcopenia	24	2 (8.3%)^b^	22 (91.7%)

Data are presented as n (%). Pairwise comparisons used two-sided Fisher’s exact tests with Bonferroni correction pairwise comparisons. Groups without a shared superscript letter differed significantly (adjusted P< 0.05).

**Figure 3:**
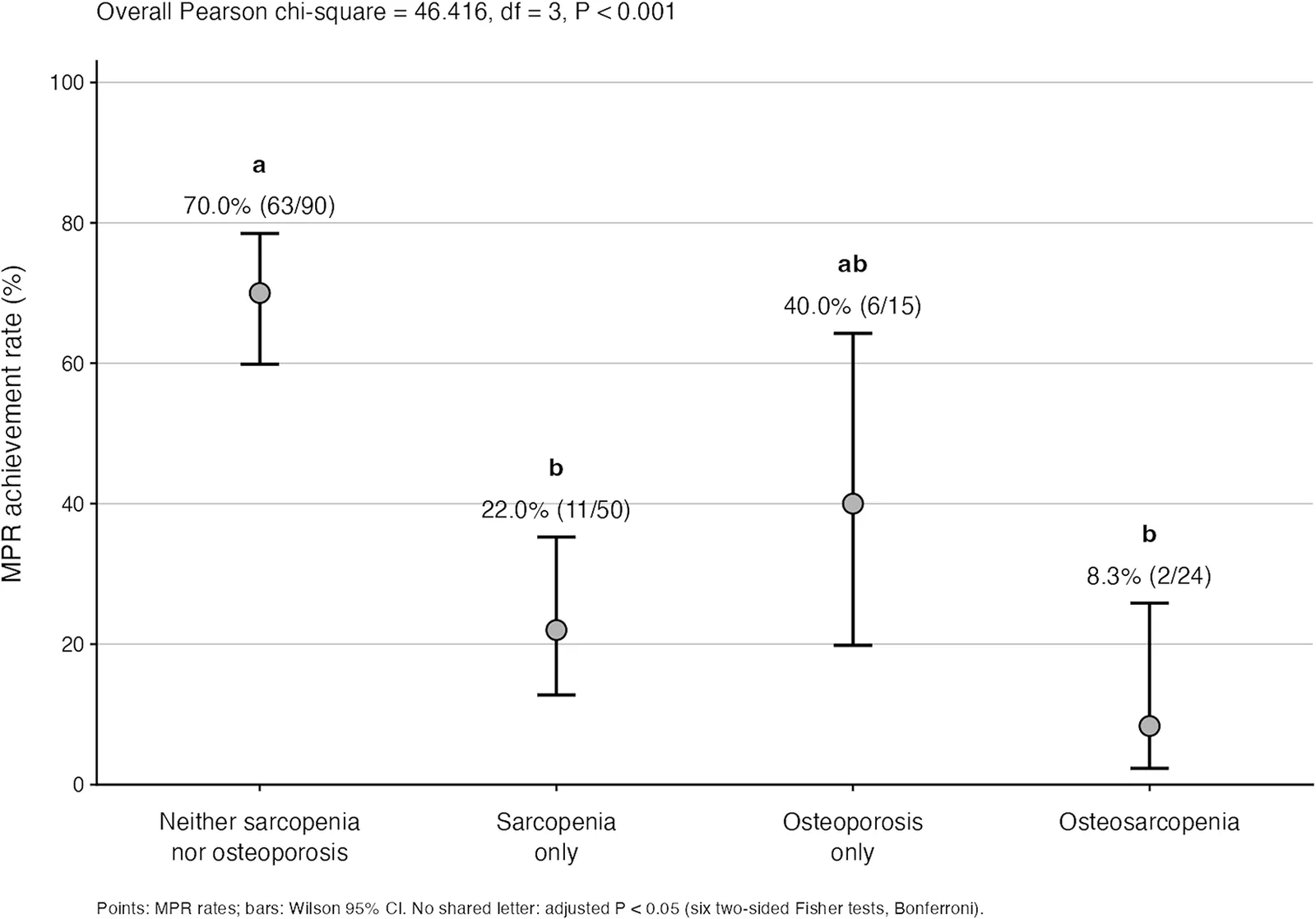
Major pathological response rates according to sarcopenia and osteoporosis status. Points represent observed major pathological response rates, and error bars indicate Wilson 95% confidence intervals. Group sizes were 90, 50, 15, and 24 for neither condition, sarcopenia only, osteoporosis only, and osteosarcopenia, respectively. Groups without a shared letter differed significantly (adjusted P< 0.05).

### Feature selection and model construction

Achievement of major pathological response was defined as the positive outcome (MPR = 1). The predicted probability of achieving MPR was calculated as:


$$P\left(\text{MPR}=1\right)=\frac{1}{1+\exp\left[-\left(0.385-1.687X_{1}-1.006X_{2}-2.216X_{3}-1.330X_{4}+0.108X_{5}\right)\right]},$$

Where *X_j_* denotes its corresponding value in the final model. Intercept, regression coefficients, odds ratios (ORs), 95% confidence intervals, and P values of the final LASSO–Firth model are presented in **Table 4** and **Figure 4**. The reported ORs were adjusted for the other predictors included in the final model: tumor grade, comorbidity, LVI, sarcopenia, and IMAT area percentage. In the multivariable Firth logistic regression analysis, higher tumor grading, comorbidity, LVI, and sarcopenia were associated with lower odds of achieving MPR, whereas a higher IMAT area was associated with increased odds of achieving MPR. Osteoporosis was not significantly associated with MPR. A nomogram based on the final model was constructed to provide individualized estimates of the probability of achieving MPR (**Figure 5**).

**Table 4:** Results of the final LASSO–Firth model.

Factor	Variable	β	OR	95% CI	P value
Intercept	β_0_	0.385	1.470	0.246–8.792	0.673
Tumor grading	X_1_ (β_1_)	−1.687	0.185	0.101–0.340	<0.001
Comorbidity	X_2_ (β_2_)	−1.006	0.366	0.145–0.925	0.034
LVI	X_3_ (β_3_)	−2.216	0.109	0.026–0.452	0.002
Sarcopenia	X_4_ (β_4_)	−1.330	0.265	0.108–0.647	0.004
IMAT area (%)	X_5_ (β_5_)	0.108	1.114	1.022–1.215	0.014

MPR, major pathological response; OR, odds ratio; CI, confidence interval; LVI, lymphovascular invasion; IMAT, intermuscular adipose tissue.

**Figure 4:**
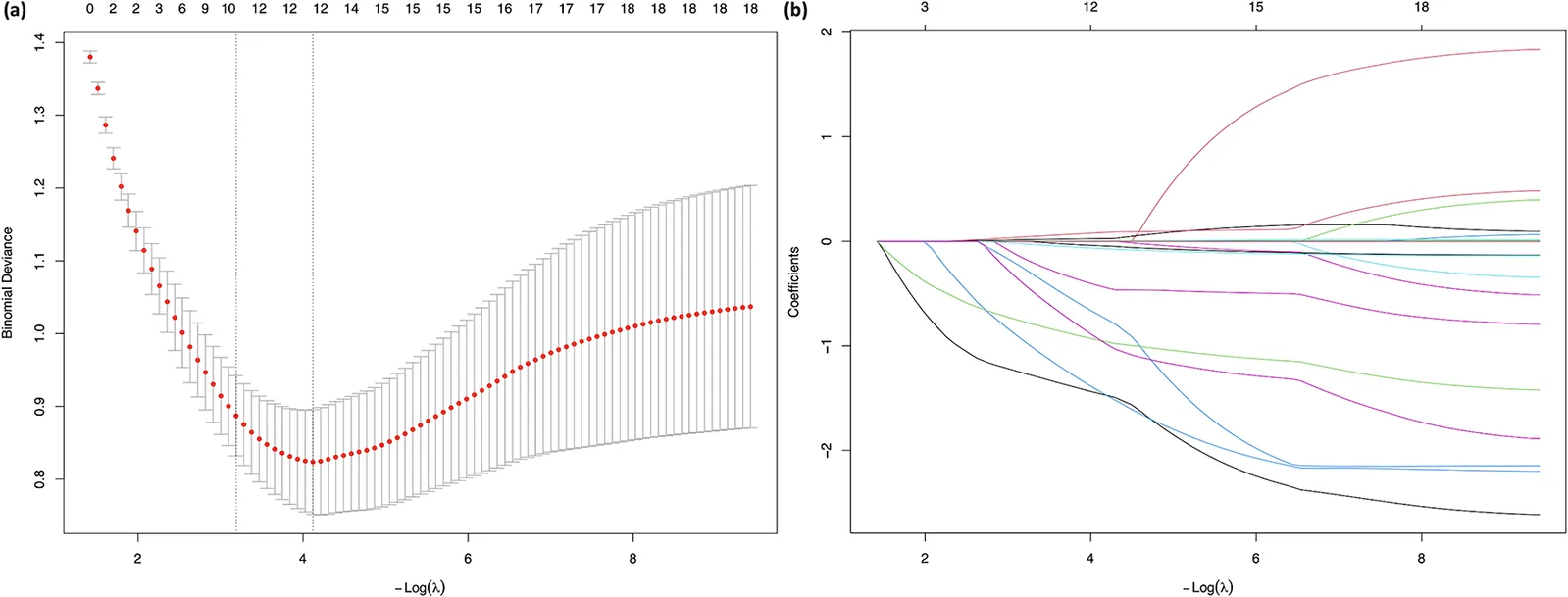
Feature selection using LASSO penalized logistic regression for predicting. **(A)** Five-fold cross-validation curve used to determine the optimal penalty parameter (*λ*). **(B)** LASSO coefficient profiles with MPR achievement coded as the positive outcome.

**Figure 5:**
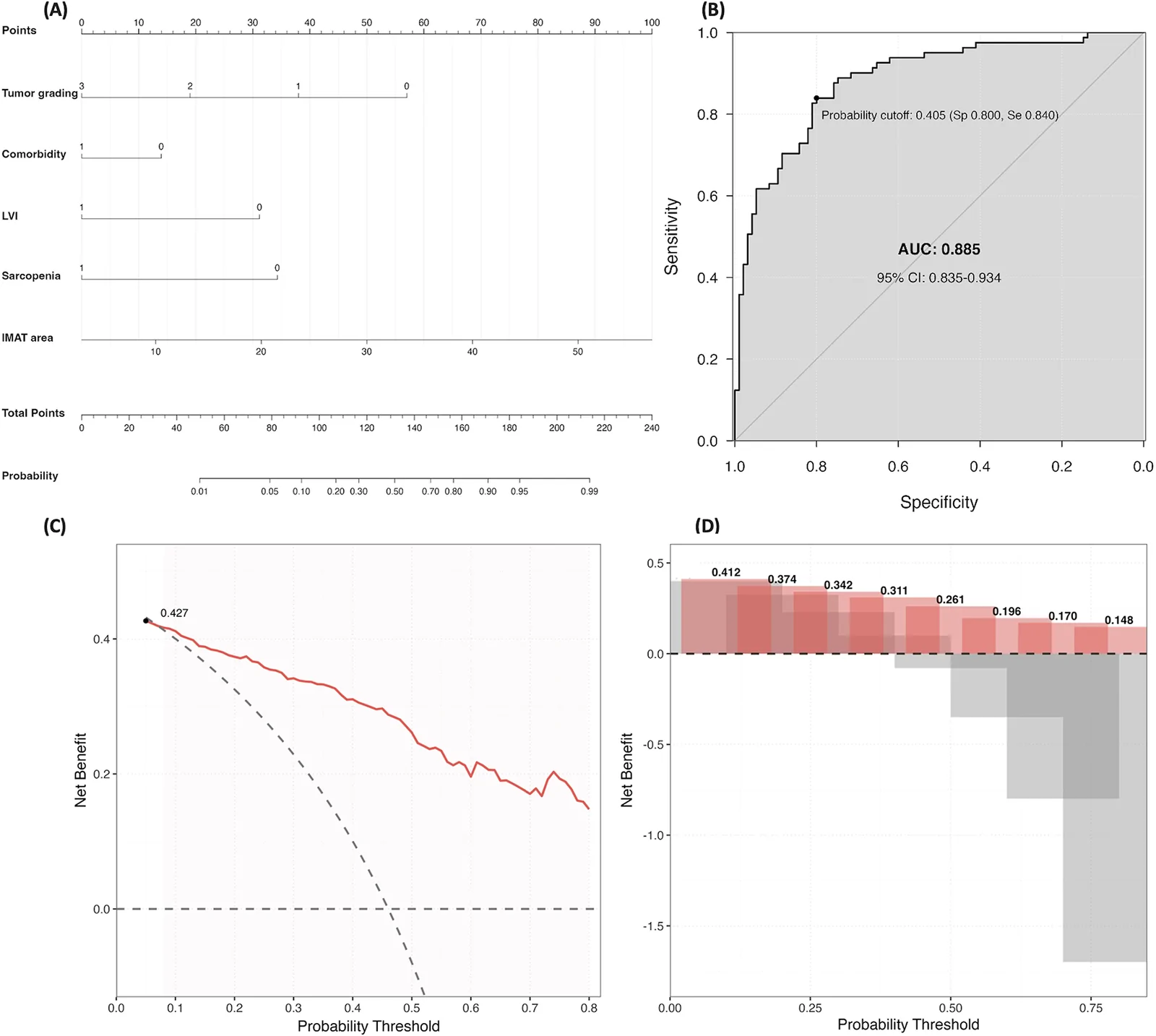
Internal validation and clinical utility of the LASSO–Firth model. **(A)** Nomogram derived from the final model refitted using the complete-case cohort. **(B)** ROC curve based on the pooled cross-fitted recalibrated out-of-bag predictions from 500 stratified bootstrap iterations. **(C)** Decision curve analysis of the cross-fitted recalibrated model. **(D)** Comparison of net benefit at selected probability thresholds.

### Model performance evaluation

Using the aggregated out-of-bag predictions from the repeated-bootstrap procedure, the cross-fitted recalibrated model achieved an AUC of 0.885 (95% CI, 0.835–0.934) (**Figure 5**) and a Brier score of 0.136. At the optimal probability threshold of 0.405, the accuracy, sensitivity and specificity were 0.818, 0.840 and 0.800, respectively. The calibration intercept and slope were −0.009 and 0.960, respectively, and the Hosmer–Lemeshow test showed no substantial evidence of lack of fit (χ²=10.707, df=8, P = 0.219).

Decision curve analysis (**Figure 5**) showed that the cross-fitted recalibrated model yielded greater net benefit than either the treat-all or treat-no strategy across threshold probabilities of 0.08–0.80. These findings support its potential clinical utility for predicting the achievement of MPR.

### Sensitivity analyses of IMAT

In the primary analysis, IMAT area was positively associated with the achievement of MPR (OR = 1.120, 95% CI, 1.044–1.218, P<0.001). After adjustment for BMI, IMAT remained significantly associated with the achievement of MPR (adjusted OR = 1.119, 95% CI, 1.042–1.218, P<0.001). The association also remained significant after additional adjustment for visceral adipose tissue area (OR = 1.118, 95% CI, 1.041–1.217, P<0.001) or subcutaneous adipose tissue area (OR = 1.106, 95% CI, 1.025–1.207, P = 0.007). However, when BMI, visceral adipose tissue, and subcutaneous adipose tissue area were included simultaneously, the association was attenuated and no longer statistically significant (OR = 1.071, 95% CI, 0.982–1.179, P = 0.127).

## Discussion

NACI has emerged as a promising treatment option for locally advanced ESCC, although pathological responses vary substantially among patients. This study evaluated the associations of sarcopenia, osteoporosis and CT-derived body-composition parameters with MPR achievement. MPR rates were 70.0%, 40.0%, 22.0%, and 8.3% in the control, osteoporosis-only, sarcopenia-only, and osteosarcopenia groups, respectively. Multivariable Firth logistic regression showed that lower tumor grade, absence of comorbidities, absence of LVI, absence of sarcopenia, and a higher IMAT area percentage were associated with greater odds of achieving MPR. In contrast, osteoporosis was not independently associated with MPR (P = 0.130). The combined clinical and CT body-composition model showed good internally validated performance.

The model was internally validated in 179 complete cases using 500 repeated stratified bootstrap iterations with out-of-bag prediction and five-fold cross-fitted recalibration. The cross-fitted recalibrated model achieved an AUC of 0.885 (95% CI, 0.835–0.934). At a probability threshold of 0.405, accuracy, sensitivity, and specificity were 0.818, 0.840, and 0.800, respectively. The Brier score was 0.136, with a calibration intercept of −0.009 and a slope of 0.960. The Hosmer–Lemeshow test showed no evidence of substantial lack of fit (P = 0.219). Decision curve analysis showed greater net benefit than the treat-all and treat-no strategies across threshold probabilities of 0.08–0.80. These findings support the model’s discrimination, calibration, and potential clinical utility within the present cohort. Nevertheless, independent external validation remains necessary.

The prevalence rates of osteoporosis, sarcopenia, and osteosarcopenia in this cohort were 21.7%, 41.3%, and 13.4%, respectively. Tumor-related dysphagia, inadequate nutritional intake, negative nitrogen balance, older age, and comorbidities may collectively contribute to muscle and bone loss. Previous studies have also reported a high prevalence of osteosarcopenia in colorectal, pancreatic, and hepatocellular cancers (12, 13, 17). These observations highlight the importance of assessing muscle and bone health in patients with ESCC.

MPR achievement differed significantly among the four groups. Before correction for multiple comparisons, osteosarcopenia and osteoporosis alone were associated with lower odds of achieving MPR. However, the difference between the osteoporosis-only and control groups was no longer significant after Bonferroni correction. The interaction between sarcopenia and osteoporosis was also not significant (OR = 1.05, P = 0.96). Moreover, osteoporosis was not independently associated with MPR in the multivariable Firth model (P = 0.130). The small osteoporosis-only and osteosarcopenia groups may have limited statistical power and the precision of the effect estimates. Therefore, the present findings neither establish osteoporosis as an independent factor associated with MPR nor exclude a potential interaction. Previous evidence suggests that bone loss may precede substantial muscle loss (18). Cancer-related inflammation and dysregulated bone metabolism may also affect both skeletal health and tumor progression (19, 20). However, these potential mechanisms require further investigation.

Sarcopenia was associated with lower odds of achieving MPR, whereas a higher IMAT area percentage was associated with greater odds of MPR. Sarcopenia may reflect the combined effects of nutritional depletion, systemic inflammation, reduced functional reserve, and impaired treatment tolerance. Zhao et al. reported that changes in the skeletal muscle index during neoadjuvant immunochemotherapy were associated with pathological response and postoperative complications in 85 patients with ESCC (21). However, den Boer et al. found no association between muscle index changes during neoadjuvant chemoradiotherapy and postoperative complications or overall survival in 126 patients (22). Differences in treatment regimens, body-composition measurements, and clinical endpoints limit direct comparisons with our MPR-focused findings. Previous studies have associated a larger subcutaneous adipose tissue area with better outcomes in some patients with cancer (23). These observations have contributed to the concept of the “obesity paradox” (24, 25). Greater adipose reserves may help patients tolerate treatment-related energy demands, although this explanation remains speculative (26). Onishi et al. reported that a transition to low visceral fat mass during neoadjuvant chemotherapy was associated with poorer survival in patients with esophageal cancer (27). Their earlier study linked concurrent decreases in psoas muscle index and increases in visceral fat to more postoperative complications and longer hospitalization (28). These findings suggest that adipose tissue associations should be interpreted alongside muscle status and treatment-related changes. However, these studies evaluated visceral fat rather than IMAT and therefore do not directly validate the IMAT–MPR association observed in our cohort. After adjustment for BMI, IMAT remained associated with MPR achievement (OR = 1.119; 95% CI, 1.042–1.218; P< 0.001). However, simultaneous adjustment for BMI, visceral adipose tissue area, and subcutaneous adipose tissue area attenuated this association (OR = 1.071; 95% CI, 0.982–1.179; P = 0.127). This attenuation may reflect correlations and shared information among body-composition measurements. Therefore, a higher IMAT area percentage should not be interpreted directly as protective. Its independent contribution requires confirmation in larger cohorts with documented weight-loss histories and standardized measurements of individual adipose compartments.

Tumor grade and LVI were associated with MPR after adjustment for the other predictors retained in the final model. This finding is consistent with previous studies linking tumor invasion depth, histological grade, and lymph-node involvement to response after neoadjuvant therapy (29, 30). LVI may reflect greater tumor aggressiveness and a more complex tumor microenvironment. Inflammation-mediated abnormal angiogenesis may impair tissue perfusion and immune-cell infiltration, thereby weakening immune surveillance and therapeutic efficacy (31–33). We also evaluated inflammatory and nutritional markers, including PNI, NLR, LMR, and PLR. However, these variables were not retained in the final model, and their potential roles require evaluation in larger cohorts.

MPR was selected as the primary outcome rather than pCR. Previous studies reported a pCR rate of approximately 27.4% after NACI in ESCC, compared with an MPR rate of 45.2% (3). Unlike pCR, which requires the complete absence of viable tumor cells, MPR also captures substantial but incomplete tumor regression. MPR may therefore provide a more sensitive measure of pathological response to NACI. Previous evidence has identified MPR as an independent prognostic factor and a potential surrogate endpoint for survival in ESCC (34, 35). MPR has also been associated with progression-free and overall survival (36, 37). Accordingly, MPR represents a clinically relevant endpoint for evaluating pathological response to NACI.

All patients received the same NACI regimen comprising tislelizumab, albumin-bound paclitaxel, and nedaplatin, which reduced treatment-related heterogeneity. Our study complements this literature by evaluating CT-defined sarcopenia, osteoporosis, and IMAT area percentage in relation to MPR under a uniform NACI regimen. However, the identified factors and model performance may be regimen specific. The findings may not apply to patients receiving other PD-1 inhibitors or chemotherapy backbones. Further validation in independent multicenter cohorts receiving different immunochemotherapy regimens is therefore required.

Our findings further emphasize the importance of muscle, nutritional, and bone health during treatment for ESCC. In addition to routine nutritional support, structured physical and cardiopulmonary exercise programs may help preserve muscle mass and ameliorate sarcopenia (38). Adequate calcium and vitamin D intake may also support bone metabolism and help prevent osteoporosis (39). However, this study did not evaluate the effects of these interventions. Their associations with MPR therefore require prospective investigation.

This study has several limitations. First, the retrospective single-center design may have introduced selection bias and limits the generalizability of our findings. Differences in patient characteristics, clinical practices, imaging protocols, and treatment regimens across institutions may affect model performance. Although repeated bootstrap validation reduced model optimism, the lack of independent external validation limits assessment of its generalizability. Prospective multicenter validation is therefore required. Second, the small osteoporosis-only and osteosarcopenia groups limited statistical power, particularly for detecting an interaction. Therefore, the group comparisons should be interpreted cautiously and validated in larger cohorts. Third, CT image segmentation relied on manual delineation by experienced radiologists, which was time-consuming and potentially susceptible to interobserver and intraobserver variability. Although discrepancies were adjudicated by a third senior radiologist, some observer-related bias may remain. Advances in automated volumetric segmentation may improve reproducibility, streamline the workflow, and minimize human error.

## Conclusion

In this retrospective single-center cohort of ESCC patients receiving tislelizumab-based NACI, sarcopenia and IMAT area percentage were associated with MPR achievement. A model integrating clinicopathological characteristics and CT-derived body-composition parameters was developed and internally validated. External validation and prospective evaluation are needed to establish its generalizability and clinical utility.

## Data Availability

The data analyzed in this study is subject to the following licenses/restrictions: Further inquiries can be directed to the corresponding author. Requests to access these datasets should be directed to Suying Wu, wusuying0594@126.com.
